# Autistic Adults May Be Erroneously Perceived as Deceptive and Lacking Credibility

**DOI:** 10.1007/s10803-021-04963-4

**Published:** 2021-03-17

**Authors:** Alliyza Lim, Robyn L. Young, Neil Brewer

**Affiliations:** College of Education, Psychology, and Social Work, Flinders University, GPO Box 2100, Adelaide, South Australia 5001 Australia

**Keywords:** Autism, Perceptions, Deception, Credibility

## Abstract

We hypothesized that autistic adults may be erroneously judged as deceptive or lacking credibility due to demonstrating unexpected and atypical behaviors. Thirty autistic and 29 neurotypical individuals participated in video-recorded interviews, and we measured their demonstration of gaze aversion, repetitive body movements, literal interpretation of figurative language, poor reciprocity, and flat affect. Participants (*N* = 1410) viewed one of these videos and rated their perception of the individual’s truthfulness or credibility. The hypothesis was partially supported, with autistic individuals perceived as more deceptive and less credible than neurotypical individuals when telling the truth. However, this relationship was not influenced by the presence of any of the target behaviors, but instead, by the individual’s overall presentation.

**Supplementary Information:**

The online version contains supplementary material available at 10.1007/s10803-021-04963-4.

## Autistic Adults May Be Erroneously Perceived as Deceptive and Lacking Credibility

Interpersonal judgments frequently involve an attempt to determine if another individual is trustworthy and believable or perhaps lying or attempting to deceive in some way. Such judgments are important components of the task that confronts judges and jurors, but they are also required in a variety of other contexts. For example, is the salesperson trying to sell you a car telling the truth about the history of the car? Is the politician making promises during an election campaign someone you can trust? When your adolescent son or daughter tells you where they have been until 3 a.m., can you rely on what they say?

How then do you spot a liar? If your answer was that liars fidget and avoid eye contact, you would not be alone. An international study conducted by the The Global Deception Research Team (2006) found that gaze aversion and fidgeting are the two behaviors most commonly perceived as indicating deception. It is both coincidental and unfortunate that, to varying degrees, these behaviors form part of the diagnostic criteria for, and may characterize, individuals with autism spectrum disorder (ASD). Research findings appear to be unanimous that people on the autism spectrum demonstrate more gaze aversion than their neurotypical peers (Doherty-Sneddon et al., 2013; Klin et al., 2002; Riby & Hancock, 2008). Furthermore, the repetitive patterns of behavior often demonstrated by people with autism, which include repetitive movements such as rocking, pacing, finger flicking, and hand or foot tapping (Cunningham & Schreibman, 2008), have been associated with perceptions of dishonest behavior. We hypothesized that the marked similarity between perceived indicators of deception and common autistic behaviors may cause people on the autism spectrum to be judged as more deceptive than their neurotypical peers when telling the truth.

### Behavioral Markers of Deception

Research on behavioral markers of deception has demonstrated that, contrary to popular belief, there is no evidence that gaze aversion and body movements are indicative of deception (DePaulo et al., 2003; Mann et al., 2012, 2013; Sporer & Schwandt, 2007; Vrij, 2019). In fact, a recent study by Luke (2019) suggests that there is currently insufficient information in the literature to conclude the existence of *any* reliable behavioral cue to deception. Yet, gaze aversion and body movements remain pervasive stereotypes of cues to deception, held even by presumed expert lie detectors such as police officers, customs officers, prosecutors, and judges (Akehurst et al., 1996; Bogaard & Meijer, 2018; Bogaard et al., 2016; Delmas et al., 2019; Dickens & Curtis, 2019; Strömwall & Granhag, 2003; Vrij & Semin, 1996; Vrij et al., 2006).

### Why Do We Rely on Unreliable Cues?

Attribution theories provide a widely accepted theoretical framework to explain the way in which behavioral cues are used in the formation of deception and credibility judgments. Attribution theories suggest that individuals are naturally driven to understand the causes of events and will use their knowledge of an individual’s personal and situational characteristics to attribute an explanation for observed behaviors (Kelley, 1967). For example, the expectancy violations theory (Burgoon, 1983; Burgoon & Hale, 1988; Burgoon & Jones, 1976; Burgoon et al., 1989) suggests that people hold expectations about how others behave in social interactions. When an individual violates these expectancies, the attention of their communication partner is drawn away from the interaction and toward the unexpected behavior. Observers then interpret this behavior in light of personal and situational characteristics to form an appraisal of the violation; if the behavior is interpreted as a negative violation, it results in unfavorable communication outcomes (Burgoon & Hale, 1988).

Based on this premise, Levine et al. (2000) and Bond et al. (1992) proposed the norm-violation model and expectancy-violation model of deception judgments, respectively. These models state that, while nonverbal behaviors that are expected or normative are accepted at face value, nonverbal behaviors that are unexpected (Bond et al., 1992) or atypical (Levine et al., 2000) demand an explanation and raise suspicion about the sender’s intentions. They argue that it is not the specific behaviors of gaze aversion or fidgeting per se that leads to deception judgments but the fact that these behaviors violate social norms and expectations, therefore prompting the observer to infer deception as a means of explaining the behavior (Bond et al., 1992; Levine et al., 2000).

In instances where multiple possible causes for a behavior exist, Kelley (1971) proposes that the observer employs a discounting principle—as more plausible reasons for the behavior come to light, the importance of each individual cause is diminished. The relative importance of a particular cause is evaluated based on the number and perceived significance of all possible alternative explanations. For example, if an observer witnesses an individual fidgeting and avoiding eye contact while testifying in court, the observer may assume that the individual is being deceptive. However, if the observer is aware that the individual has been diagnosed with autism, the observer may be less likely to attribute deception as the cause of the behavior. Given autism is largely unidentifiable by physical appearance, observers are unlikely to be aware of an individual’s diagnosis and unlikely to expect behaviors such as gaze aversion and fidgeting. Therefore, when these, or other autistic behaviors, are displayed, there is an incongruence between the observer’s expectations and the individual’s behavior, leading the observer to search for possible explanations for the behavior such as deception and low credibility. Thus, characteristics of autism that are both atypical and incongruent with social norms may be viewed as indicative of deception.

### Deception and Credibility

A concept that is closely related to deception is source credibility. In a study of the relationship between perceived deception and source credibility, O'Sullivan (2003) proposed that the performance of human lie detectors is subject to the fundamental attribution error: that is, the tendency to overestimate the importance of dispositional traits of an individual and to underestimate the importance of situational factors (Ross & Nisbett, 1991). O'Sullivan (2003) explained that, because individuals are often unable to distinguish between trait truthfulness (whether the individual is trustworthy) and state truthfulness (whether the individual is telling the truth in that specific instance), they tend to assume that trustworthy individuals always tell the truth and untrustworthy individuals are always deceptive. Therefore, to understand perceptions of deception, it would also be beneficial to consider factors that influence perceptions of credibility, as these constructs are closely related.

One cue used to infer credibility is emotional expression (Heath, 2009; Kaufmann et al., 2003; Melinder et al., 2016; Wessel et al., 2013). A meta-analysis of 20 studies by Nitschke et al. (2019) found that the level of distress displayed by rape victims significantly influenced the degree to which they were perceived as credible complainants, with those who displayed congruent emotions (e.g., crying) being perceived as more credible than those who displayed incongruent (e.g., smiling) or neutral emotions (e.g., flat affect). This tendency to rely on emotional displays in forming credibility judgments once again raises the question of whether autistic individuals, who are known to display reduced emotional expressivity (Stagg et al., 2014; Zantinge et al., 2019), would be perceived as less credible than their neurotypical peers.

### Autistic Behaviors and Perceptions of Deception and Credibility

Given that many forms of social interaction involve some degree of impression formation, misinterpretation of autistic behaviors has the potential to cause detrimental consequences for people on the autism spectrum (see Denault & Jupe, 2018; Porter & ten Brinke, 2009; Vrij & Turgeon, 2018), particularly in situations such as interactions with the criminal justice system. It is, therefore, important to understand the influence of autistic behaviors on the formation of deception and credibility judgments. In addition to examining gaze aversion, repetitive body movements, and flat affect, this study examines two characteristic behaviors of people on the autism spectrum that could potentially be viewed as indicative of deception or low credibility: poor reciprocity and literal interpretation of figurative language. Although little attention has been paid to these behaviors in the deception and credibility literature, their atypical and unexpected nature may also cause them to be interpreted negatively.

As part of their difficulty with socioemotional reciprocity, autistic individuals are often known to have trouble maintaining two-way conversation (American Psychiatric Association, 2013). Due to impairments in their ability to understand the perspective of their conversation partner, autistic individuals may talk exclusively about their own interests, failing to recognize that this may not be of interest to the listener (Chin & Bernard-Opitz, 2000). Autistic individuals may also be less responsive to cues for turn-exchange and have difficulty conforming to appropriate norms for the timing and latency of turn-taking (Paul et al., 2009). Furthermore, research has demonstrated that autistic individuals perform more poorly than their neurotypical counterparts on tasks that require the understanding of figurative language (Cheung et al., 2019; Saban-Bezalel et al., 2019; Saban-Bezalel & Mashal, 2019).

Turn-taking is a central component of any social conversation and there is evidence that children are able to make predictions about the turn structure of a conversation from as early as two years of age (Casillas & Frank, 2017). Likewise, studies on the use of figurative language have shown that children aged eight to 10 years are able to understand and use figurative language (Pollio & Pollio, 1974) and that by age 11, children are able to identify the communicative intent behind a range of figurative statements (Demorest et al., 1983). This suggests the expectation that, by adulthood, one would have the ability to both predict and finish their turn in a social conversation, as well as be proficient in the use of figurative language. Thus, when individuals exhibit poor reciprocity or respond inappropriately to figurative language, this expectation is violated. In the absence of any apparent intellectual disability, language impairment, or cultural differences, observers may attribute the behavior to deception or low credibility with the individual seen as trying to avoid or change the subject of conversation.

### The Study

This study examined the relationship between autism, autistic behaviors, and perceptions of deception and credibility. We hypothesized that autistic individuals would be more likely than neurotypical individuals to be judged as deceptive and lacking credibility, and that this difference would be attributable to higher levels of gaze aversion, repetitive body movements, literal interpretation of figurative language, poor reciprocity, and flat affect displayed by autistic individuals.

We also hypothesized that the relationship between ASD diagnosis and perceived deception and credibility would be moderated by the knowledge that the target individual is on the autism spectrum. This hypothesis is not only consistent with Kelley’s (1971) discounting principle but also with emerging evidence that providing information on an individual’s ASD diagnosis results in more positive interpersonal judgments (Maras, Crane, et al., 2019; Maras, Marshall, et al., 2019; Matthews et al., 2015; Sasson & Morrison, 2019). For example, Maras, Marshall, et al. (2019) found that mock jurors who were informed that a defendant was autistic perceived the defendant as more honest and less guilty than those who were not informed of his diagnosis. Follow-up qualitative analyses revealed that, consistent with attribution theories, mock jurors who were told that the defendant was autistic were more likely to attribute his inappropriate behaviors to his autism, whereas mock jurors who were not given any information on ASD diagnosis reported that the defendant’s aggressive behaviors, body language, and gaze aversion led them to believe that he was being deceptive to protect his own interests (Maras, Marshall, et al., 2019).

Although gaze aversion, repetitive body movements, literal interpretation of figurative language, poor reciprocity, and flat affect are generally considered to be characteristic behaviors of autistic individuals and are reflected in diagnostic criteria, there are deficiencies in our knowledge base regarding the prevalence of these behaviors among autistic adults. Much research in this area has been conducted among children and adolescents rather than with adults. As the specific behavioral manifestations of ASD diagnostic criteria may vary over the lifespan (Fecteau et al., 2003; Georgiades et al., 2017), it is possible that the current literature does not reflect the degree to which these behaviors are displayed in an adult population. Furthermore, few studies conducted with autistic adults have measured the behaviors through direct observation, with research relying primarily on self-report or informant-report measures. Therefore, to test our hypotheses, we recruited autistic and neurotypical individuals to participate as stimuli in short-video recorded interviews and measured the degree to which they displayed each of the target behaviors. Participants were then randomly allocated to view one of these videos and to rate the extent to which they believed the target individual was deceptive or credible.

## Method

### Participants and Design

Participants were 1726 adults recruited via the online crowdsourcing platform TurkPrime (Litman et al., 2017). Three hundred and sixteen participants failed to pass one or more attention checks (e.g., “Please spell the word ‘WORLD’ backwards”), and their data were excluded. The final sample consisted of 1410 participants (853 female), ranging in age from 18 to over 85 years (*M* = 41.13). As there is evidence that judgments of deception are influenced by perceptions of credibility (George et al., 2014; O'Sullivan, 2003), a between-subjects design was used to avoid potential carry-over effects: each participant was randomly allocated to view only one of the stimulus videos and to complete only one measure of either the target individual’s truthfulness (*n* = 713) or credibility (*n* = 697). A priori sample size estimations using G*Power 3.1 (Faul et al., 2007) indicated that a minimum sample of 725 participants would be required to detect a small effect at an alpha level of 0.05 in a multiple regression model with seven predictor variables. As participants completed only one measure of either deception or credibility, a total sample of 1450 was targeted.

## Materials

### Stimulus Materials

#### Stimulus Development

Thirty-one autistic individuals (nine female) and 29 neurotypical individuals (15 female) ranging in age from 18 to 66 years (*M* = 29.62, *SD* = 11.57) were involved in the production of stimulus videos for this study. A breakdown of the demographics of the target individuals can be found in Supplemental Materials (p. 1). Twenty-one autistic individuals were recruited from a university autism database of individuals on the autism spectrum who reside in the same region as the university and have indicated interest in participating in research projects (the majority of individuals on this database were originally recruited through a mail-out by the local autism association). The remaining ten autistic individuals were recruited through an advertisement at a local psychology practice that specializes in working with people on the autism spectrum. All 31 individuals reported receiving a formal diagnosis of autism from a registered diagnostician. To access support services from the local autism association, individuals must have received a diagnosis of autism from at least two independent registered diagnosticians (i.e., speech pathologist, psychologist, pediatrician, or psychiatrist) or from a registered multidisciplinary team. None of the individuals recruited were suspected to have an intellectual disability; this was supported by records that all individuals recruited from the university autism database had a Wechsler Abbreviated Scale of Intelligence—Second Edition (WASI-II; Wechsler, 2011) intelligence quotient (IQ) above 85. Specific data on socioeconomic status and educational attainment levels of the autistic sample were not recorded. The 29 neurotypical individuals were all current university students who reported that they did not have a diagnosis of autism and were also assumed to have IQ levels above 85.

Given the heterogeneity of autism (Jeste & Geschwind, 2014; Lenroot & Yeung, 2013; Martinez-Murcia et al., 2017), we recognized that there would likely be significant variability in the demonstration of these behaviors. Although a larger sample would more likely capture this variability and provide a more powerful test of behavioral differences between the two stimulus groups, the recruitment of autistic participants who are willing to attend a video-recorded interview that will subsequently be used as stimulus material poses a considerable barrier. Nevertheless, in accordance with ASD diagnostic criteria, it was assumed that these behaviors would be more prevalent in the autistic than the neurotypical individuals.

Each individual attended a session at a psychology lab and was informed that the purpose of the session was to create videos that would be used in a research study on deception. They were told that they would be asked to complete a computer task and to then participate in a short video-recorded interview. Prior to the commencement of the task, the individuals were shown an envelope and told that it contained $20. They were informed that: “People who watch this video will be told that you may have taken this money but are trying to convince the interviewer that you did not. They will be told that (1) participants who took the money but could successfully convince the interviewer that they did not would receive $50 for participating in the study, (2) participants who took the money but were caught by the interviewer as lying would only receive $10 for participating in the study, and (3) participants who chose not to take the money would receive $20 for participating in the study, irrespective of whether they were judged as lying or being truthful. After watching the video, they will be asked to indicate whether they think you were telling the truth. However, in reality, you are not to take the money in the envelope. In the interview, it is critical that you answer all questions truthfully.” The individuals were then left to complete the computer task, after which they were interviewed by a separate researcher. This interview was video recorded.

The individuals were asked questions about the envelope in the room (e.g., “In the room, there was an envelope. Did you see this envelope?”), what they did (e.g., “Can you describe what you did after the researcher left the room?”), and whether they took the money (e.g., “Did you take the money that was in the envelope?”). The video from one autistic individual was excluded as the truthfulness of his responses could not be ascertained, resulting in a final sample of 30 autistic individuals (nine female) and 29 neurotypical individuals (15 female). Fourteen individuals (seven autistic individuals and seven neurotypical individuals) provided one or more responses in the interview that were factually incorrect (e.g., saying that they had not been told about the different payment outcomes). However, as these responses were likely the result of inattention, rather than the desire to purposefully mislead the interviewer, they were not considered to be deceptive for the purpose of this study, with deception being defined as “intentionally, knowingly, and/or purposely misleading another person” (Levine, 2014, p. 397). All individuals in the final sample correctly reported that they had not taken the money. The videos ranged in duration from 95 to 290 s (*M* = 132, *SD* = 33).

#### Behavioral Analysis

The videos were analyzed and coded for the presence of each of the five target behaviors by a research assistant who was not involved in any other aspect of this study and was blind to both the purpose of the study and group membership of the target individuals. The first author also independently analyzed and coded all 59 videos. The coders were trained on a set of six videos.

The coders watched each video five times, each time independently recording the presence of gaze aversion, repetitive body movements (excluding communicative gestures), literal interpretation of figurative language, poor reciprocity, or flat affect using a behavioral coding scheme developed for this study. The order of presentation of the videos was randomized for each behavior and coder. Inter-rater reliability for the coding of gaze aversion, repetitive body movements, and poor reciprocity was then assessed using three separate two-way random, consistency, single-measures intraclass correlations (ICC), while inter-rater reliability for the coding of literal interpretation of figurative language and flat affect were assessed using Gwet’s AC1, which is less influenced by prevalence rates than Cohen’s kappa (Feinstein & Cicchetti, 1990; Wongpakaran et al., 2013). Inter-rater reliability was high for gaze aversion (ICC = 0.88), repetitive body movements (ICC = 0.87), literal interpretation of figurative language (AC1 = 0.96), and flat affect (AC1 = 0.88), but poor for poor reciprocity (ICC = 0.51). To reconcile this discrepancy, a clinician with extensive experience working with people on the autism spectrum also recorded the levels of poor reciprocity displayed using the same operationalization and coding guidelines. Disagreements in the coding of the behavior were then discussed until a consensus was reached.

#### Clinical Impression

Given that the deficits in social communication and interaction, and restricted and repetitive behaviors and interests, that characterize autism can manifest in a variety of ways, it is likely that the behavioral analysis would fail to capture the full range of behaviors displayed. Judgments of deception and credibility may be influenced by the presence of other autistic behaviors that were not accounted for, or by the overall presentation of the individual as having a condition or disability. To examine this possibility, a measure of the general impression of each target individual was also obtained.

Six trainee psychologists, who were enrolled in an accredited postgraduate clinical psychology program and blind to both the purpose of the study and group membership of the target individuals, were recruited. They independently watched each stimulus video and indicated the likelihood that the person in the video was on the autism spectrum from 1 (*extremely unlikely*) to 7 (*extremely likely*). To ensure that the raters remained blind to the purpose of the study, they also rated the target stimulus for nine other conditions (anxiety disorder, bipolar disorder, intellectual disability, language disorder, mood disorder, obsessive–compulsive disorder, personality disorder, post-traumatic stress disorder, and schizophrenia); however, these ratings were not used as part of the data analysis. Finally, raters provided an overall rating of the likelihood that the person in the video had any mental health or developmental disorder using the same scale. The order of presentation of the videos was randomized for each rater. Inter-rater reliability was then assessed using two-way random, absolute agreement, average-measures intraclass correlations (ICC). Inter-rater reliability was acceptable for clinical impression of ASD (ICC = .70) and clinical impression of any condition (ICC = .76).

#### Perceived Deception

Perceived deception was measured with two questions: “Do you think the person in the interview was telling the truth?” rated on a 6-point Likert scale from 1 (*deceptive*) to 6 (*truthful*), and “Do you think the person in the interview took the money?” rated from 1 (*yes*) to 6 (*no*). Responses were reverse-scored and summed to obtain an overall score of perceived deception ranging from two to 12, with higher scores indicating higher levels of perceived deception.

#### Perceived Credibility

Participants rated the perceived credibility of each target individual using a modified version of McCroskey and Teven’s (1999) source credibility measure. This measure comprises three dimensions, Competence, Caring, and Character, with higher scores indicating higher levels of perceived credibility. McCroskey and Teven's (1999) validation study revealed that each of the three dimensions accounted for significant variance as unique predictors of believability and likableness, and the authors argue that the use of all three individual dimensions in the operationalization of source credibility would be more appropriate than a combined score. Further details on this measure can be found in Supplemental Materials (pp. 2–3).

### Procedure

The experiments were presented through an online survey platform (Qualtrics, Provo, UT). To avoid response bias, participants were not informed of the true purpose of the study but were instead told that the study aimed to investigate deception detection accuracy. Participants were then presented with a stimulus video and asked to provide a judgment of deception or credibility. Participants were also asked to briefly describe the reasons for their impression (they were encouraged to state “unsure” if they were uncertain). Next, participants were told, “Now if you were to be told that the person in the interview has Autism Spectrum Disorder (ASD), please rate your impression of the person in the interview again in light of this new information. Characteristics of ASD include difficulties with social interaction and nonverbal communication, repetitive behaviors, and restricted interests.” They were then asked to rate their impression of the individual a second time, using the same measure they had previously completed. Finally, a funnel debriefing procedure was used to identify any possible suspicion about the deception, and participants were debriefed on the true purpose of the study.

### Statistical Analysis

**Fig. 1:**
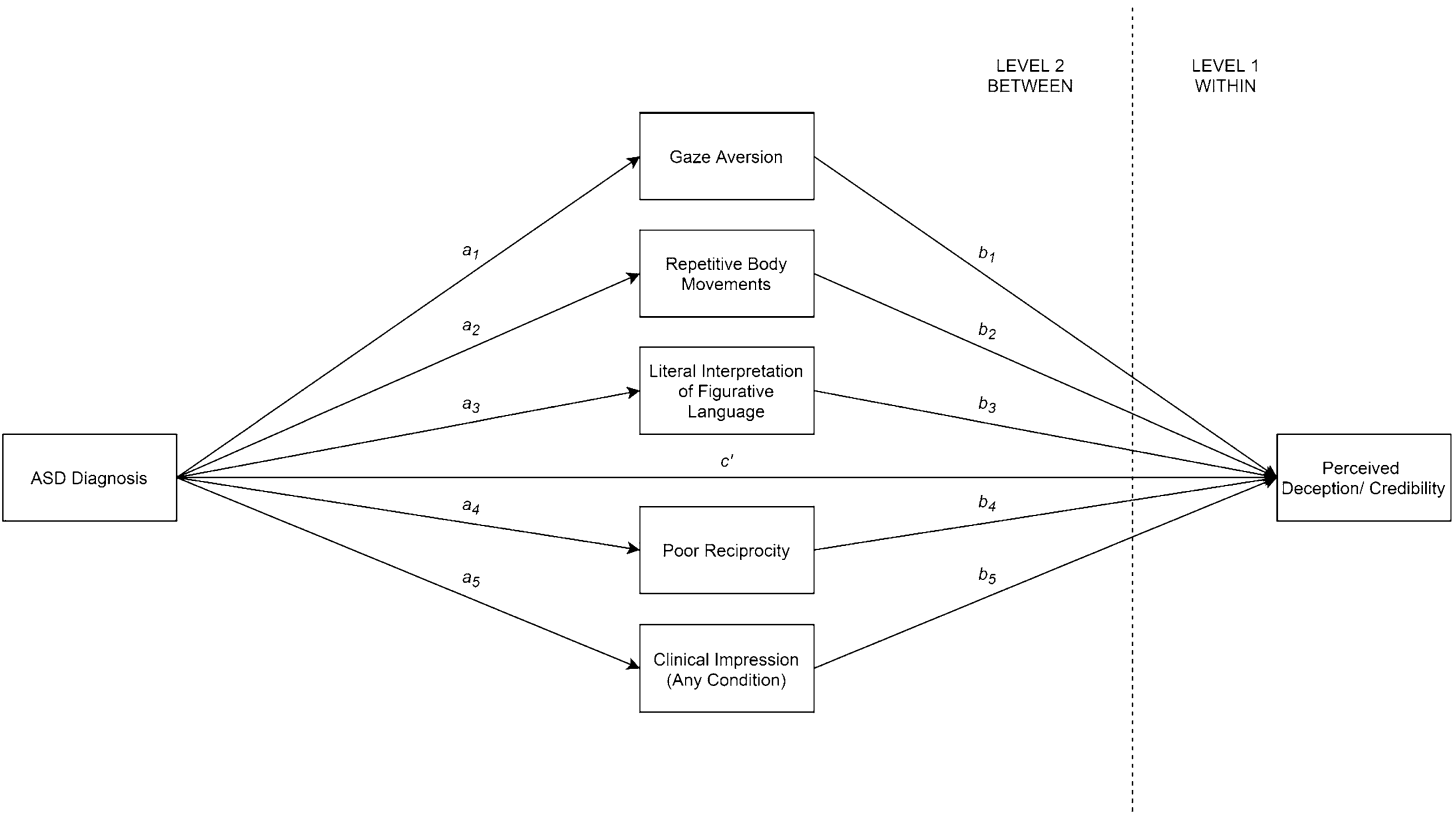
2–2–1 Multilevel mediation model between ASD diagnosis, autistic behaviors, and perceived deception or credibility

As it is unlikely that only one variable influences the predictor-outcome relationship, Preacher and Hayes (2008) advise that hypotheses involving multiple potential mediators should be considered. They recommend that when multiple mediators are involved, a multiple mediation approach (in which all mediators are included in the same model) is the most parsimonious and precise way to analyze the data (Preacher & Hayes, 2008; however, to illustrate how the strength of each mediator differed upon the inclusion of other mediators in the model, multilevel mediation analyses for each individual mediator are also presented in Supplemental Materials, pp. 4–5). Because the predictor (ASD diagnosis) and mediators (gaze aversion, repetitive body movements, literal interpretation of figurative language, poor reciprocity, flat affect, clinical impression of ASD, and clinical impression of any condition) were measured at the video level (level 2), while the outcome variables (perceived deception, perceived competence, perceived caring, and perceived character) were measured at the participant level (level 1), 2–2–1 multilevel mediation analyses were used to test the model shown in Fig. 1 using the multilevel structural equation modelling (MSEM) framework outlined by Preacher et al. (2010). Each outcome variable was examined separately. The analyses were carried out using maximum likelihood with robust standard errors estimators (MLR) in Mplus version 8.

## Results

### Behavioral Analysis

The results of the behavioral analysis and ratings of clinical impression for each group are presented in Table 1. Gaze aversion, repetitive body movements, and poor reciprocity were measured by percentage of time the behavior was displayed. Clinical impression of ASD and clinical impression of any condition were measured on 7-point Likert scales ranging from 1 (*extremely unlikely*) to 7 (*extremely likely*). The number of individuals who were coded as displaying literal interpretation of figurative language (binary scale) and flat affect (ordinal scale) are also presented.

**Table 1:** Behavioral coding of autistic behaviors and ratings of clinical impression

Autistic behavior/clinical impression	ASD (*n* = 30)		Neurotypical (*n* = 29)	
	M	SD	M	SD
Gaze aversion	35.39	13.73	29.31	11.36
Repetitive body movements	23.51	10.18	21.59	13.25
Poor reciprocity	1.79	3.42	0.80	1.98
Clinical impression (ASD)	3.15	1.01	2.11	0.64
Clinical impression (any condition)	4.21	0.96	2.93	0.64
Autistic behavior	ASD (*n* = 30)		Neurotypical (*n* = 29)	
	No. of individuals		No. of individuals	
Literal interpretation of figurative language	5		1	
Flat affect	5		3	

There was no significant difference in the demonstration of gaze aversion [*t*(57) = 1.85, *p* = .07, *d* = 0.48, 95% CI  − 0.04, 1.00], repetitive body movements [*t*(57) = 0.63, *p* = .53, *d* = 0.16, 95% CI − 0.35, 0.67], poor reciprocity [*U* = 345.00, *p* = .11, *d* = 0.36], literal interpretation of figurative language [χ^2^(1) = 2.82, *p* = .09, *d* = .45], and flat affect [χ^2^(1) = 0.50, *p* = .48, *d* = 0.18] between the autistic and neurotypical groups. However, there was considerable variability within each group (as shown by the standard deviations) and small effect sizes in the hypothesized direction were detected in gaze aversion, poor reciprocity, and literal interpretation of figurative language. Autistic individuals were rated significantly higher on clinical impression of ASD [*t*(49.20) = 4.77, *p* < .001, *d* = 1.23, 95% CI 0.68, 1.79] and clinical impression of any condition [*t*(57) = 6.00, *p* < .001, *d* = 1.56, 95% CI 0.98, 2.15] than neurotypical individuals.

### The Relationship Between ASD Diagnosis, Autistic Behaviors, and Perceived Deception and Credibility

When all mediators were included in the model, there was high multicollinearity for clinical impression of ASD (VIF = 4.11) and clinical impression of any condition (VIF = 3.64). Thus, only clinical impression of any condition was retained in the data analysis. The inter-item correlations between clinical impression of any condition and each of the five target behaviors were: .24 (gaze aversion), .04 (repetitive body movements), .08 (literal interpretation of figurative language), .30 (poor reciprocity), and .44 (flat affect). None of the target individuals displayed high levels of flat affect, only demonstrating either minimal emotional expression (*n* = 8) or appropriate emotional expression (*n* = 51). This resulted in a highly disproportionate binary outcome. Consequently, when flat affect was included in the model, the standard errors of the model parameters could not be reliably estimated. Therefore, for the purpose of hypothesis testing, flat affect was excluded from the overall model (for comparison, the results of the analysis when flat affect was included as a mediator in the model are presented in Supplemental Materials, p. 6). The results of the analysis are presented in Table 2.

**Table 2:** Multilevel mediation models between ASD diagnosis, autistic behaviors, and perceived deception and credibility

	Perceived deception		Perceived competence		Perceived caring		Perceived character	
	Estimate	95% CI	Estimate	95% CI	Estimate	95% CI	Estimate	95% CI
Total effect								
ASD diagnosis (*c*)	0.86**	**0.39, 1.33**	**−** 2.49***	**−** **3.54, −** **1.44**	**−** 0.59	**−** 1.18, 0.01	**−** 1.91*	**−** **3.33, −** **0.48**
Direct effects								
ASD diagnosis (*c’*)	0.71*	**0.14, 1.29**	**−** 0.82	**−** 2.14, 0.50	0.01	**−** 0.70, 0.72	0.32	**−** 1.49, 2.13
Mediators on ASD diagnosis								
Gaze aversion (*a*_1_)	6.07	0.77, 11.37	6.07	0.77, 11.37	6.07	0.77, 11.37	6.07	0.77, 11.37
Repetitive body movements (*a*_2_)	1.92	**−** 3.06, 6.90	1.92	**−** 3.06, 6.90	1.92	**−** 3.06, 6.90	1.92	**−** 3.06, 6.90
Literal interpretation (*a*_3_)	0.13	0.01, 0.26	0.13	0.01, 0.26	0.13	0.01, 0.26	0.13	0.01, 0.26
Poor reciprocity (*a*_4_)	1.00	**−** 0.18, 2.17	1.00	**−** 0.18, 2.17	1.00	**−** 0.18, 2.17	1.00	**−** 0.18, 2.17
Clinical impression (*a*_5_)	1.29***	**0.94, 1.63**	1.29***	**0.94, 1.63**	1.29***	**0.94, 1.63**	1.29***	**0.94, 1.63**
Outcome on mediators								
Gaze aversion (*b*_1_)	**−** 0.01	**−** 0.03, 0.02	**−** 0.004	**−** 0.05, 0.04	**−** 0.01	**−** 0.03, 0.01	**−** 0.01	**−** 0.06, 0.04
Repetitive body movements (*b*_2_)	**−** 0.02	**−** 0.04, **−** 0.001	**−** 0.03	**−** 0.07, 0.003	0.02	**−** 0.002, 0.05	**−** 0.01	**−** 0.06, 0.04
Literal interpretation (*b*_3_)	**−** 0.07	**−** 0.60, 0.46	0.94	**−** 0.64, 2.53	0.69	**−** 0.07, 1.44	1.79	**−** 0.22, 3.80
Poor reciprocity (*b*_4_)	**−** 0.11**	**−** **0.17, −** **0.04**	**−** 0.07	**−** 0.25, 0.11	**−** 0.03	**−** 0.09, 0.04	**−** 0.12	**−** 0.26, 0.03
Clinical impression (*b*_5_)	0.27	**−** 0.02, 0.55	**−** 1.28**	**−** **2.01, −** **0.54**	**−** 0.51**	**−** **0.83, −** **0.19**	**−** 1.75***	**−** **2.58, −** **0.92**
Indirect effects								
Total indirect effect (*ab*)	0.15	**−** 0.27, 0.57	**−** 1.67*	**−** **2.83, −** **0.51**	**−** 0.60	**−** 1.10, **−** 0.09	**−** 2.22**	**−** **3.52, −** **0.93**
Gaze aversion (*a*_1_*b*_1_)	**−** 0.03	**−** 0.16, 0.10	**−** 0.02	**−** 0.27, 0.23	**−** 0.05	**−** 0.18, 0.09	**−** 0.07	**−** 0.38, 0.23
Repetitive body movements (*a*_2_*b*_2_)	**−** 0.04	**−** 0.16, 0.07	**−** 0.06	**−** 0.24, 0.12	0.04	**−** 0.09, 0.17	**−** 0.03	**−** 0.14, 0.09
Literal interpretation (*a*_3_*b*_3_)	**−** 0.01	**−** 0.08, 0.06	0.13	**−** 0.13, 0.38	0.09	**−** 0.06, 0.24	0.24	**−** 0.18, 0.66
Poor reciprocity (*a*_4_*b*_4_)	**−** 0.11	**−** 0.23, 0.02	**−** 0.07	**−** 0.26, 0.12	**−** 0.03	**−** 0.10, 0.04	**−** 0.12	**−** 0.30, 0.07
Clinical impression (*a*_5_*b*_5_)	0.34	**−** 0.04, 0.73	**−** 1.64*	**−** **2.77, −** **0.51**	**−** 0.65*	**−** **1.10, −** **0.21**	**−** 2.25**	**−** **3.43, −** **1.07**
Indices of model fit	RMSEA = .04, CFI = .81, TLI = .60		RMSEA = .04, CFI = .85, TLI = .68		RMSEA = .04, CFI = .82, TLI = .63		RMSEA = .04, CFI = .84, TLI = .66	

*Note*. Estimates that are significant at the .05 level are indicated in bold

*p ≤ .05, **p ≤ .01, ***p ≤ .001

The findings revealed that autistic individuals were rated higher on perceived deception and lower on perceived competence and character compared to neurotypical individuals. As indicated by the regression coefficient of the *c* pathway, the mean rating of perceived deception for the autistic group was 0.86 points higher than that of the neurotypical group, on a scale ranging from 2 to 12. Likewise, the mean rating of perceived competence was 2.49 points lower for the autistic group than the neurotypical group (scale = 5–35), and the mean rating of perceived character was 1.91 points lower for the autistic group than the neurotypical group (scale = 6–42). To the best of our knowledge, this study is among the first to provide empirical evidence for the existence of such a relationship. There was no difference in ratings of perceived caring between the two groups.

### The Relationship Between ASD Diagnosis and Autistic Behaviors

As suggested by the earlier between-group comparison of target behaviors, ASD diagnosis did not significantly predict levels of gaze aversion, repetitive body movements, literal interpretation of figurative language, or poor reciprocity (*a*_1_*–a*_4_). However, ASD diagnosis was a significant predictor of overall clinical impression (*a*_5_).

### The Relationship Between Autistic Behaviors and Perceived Deception and Credibility

Poor reciprocity (*b*_*3*_) was a significant predictor of perceived deception but not in the expected direction: the higher the level of poor reciprocity displayed by the target individual, the less deceptive they were perceived to be. Independent of ASD diagnosis, there was no significant association between any of the autistic behaviors and perceived competence, caring, or character (*b*_1_*–b*_4_), but overall clinical presentation negatively predicted ratings of perceived competence, caring, and character (*b*_5_).

### Mediation of the Relationship Between ASD Diagnosis and Perceived Deception and Credibility

Contrary to the hypothesis, no statistically significant mediation pathways were found for the relationship between ASD diagnosis and perceived deception, and only the mediation analyses of the relationships between ASD diagnosis and perceived competence, caring, and character through clinical impression were statistically significant.

### Participant-Reported Cues Indicative of Deception and Low Credibility

After indicating their impression of the target individual’s truthfulness or credibility, participants were asked to provide a brief explanation of the reasons for their rating (participants were encouraged to state “unsure” if they were uncertain). The participants’ qualitative responses were then analyzed, and the number of participants who reported using each of the target behaviors as a cue to deception or credibility was recorded. Given that participants provided open-ended responses, it was possible for participants to indicate the use of more than one cue. Besides the target behaviors, other commonly reported cues included hesitation, smiling, and a change from baseline demeanor, and the number of participants who reported using each of these cues was also calculated. Responses that did not include any of the eight listed behaviors were classified under “others” (see Table 3). To ensure the reliability of the analysis, a research assistant also independently coded 20% of the data (*n* = 282). Inter-rater reliability using Gwet’s AC1 ranged from .93 to 1 (Gwet, 2008; Wongpakaran et al., 2013).

**Table 3:** Participant-reported cues indicative of deception and low credibility

Behavioral cue	Deception (*n* = 713)		Credibility (*n* = 697)	
	*n*	%	*n*	%
Gaze aversion	202	28.33	111	15.93
Repetitive body movements	108	15.15	67	9.61
Literal interpretation of figurative language	2	0.28	2	0.29
Poor reciprocity	33	4.63	13	1.87
Flat affect	3	0.42	4	0.57
Hesitation	57	7.99	37	5.31
Smiling/ smirking/ laughing	85	11.92	48	6.89
Inconsistent demeanor	98	13.74	38	5.45
Others	237	33.24	375	53.80
Unsure	56	7.85	93	13.34

### Knowledge of ASD Diagnosis

Using the same multilevel mediation approach previously described, we examined the relationship between ASD diagnosis, autistic behaviors, and ratings of deception and credibility after participants had been told that the target individual may have autism. There was no longer a significant total effect of ASD diagnosis on perceived deception or character; however, ASD diagnosis continued to affect ratings of perceived competence, and this relationship was influenced by overall clinical impression (see Table 4).

**Table 4:** Multilevel mediation models between ASD diagnosis, autistic behaviors, and perceived deception and credibility (with knowledge of ASD)

	Perceived deception		Perceived competence		Perceived caring		Perceived character	
	Estimate	95% CI	Estimate	95% CI	Estimate	95% CI	Estimate	95% CI
Total effect								
ASD diagnosis (*c*)	0.20	**−** 0.21, 0.60	**−** 1.57***	**−** **2.35, −** **0.79**	**−** 0.39	**−** 0.88, 0.10	**−** 0.48	**−** 1.41, 0.46
Direct effects								
ASD diagnosis (*c’*)	0.25	**−** 0.27, 0.76	**−** 0.37	**−** 1.36, 0.62	0.11	**−** 0.57, 0.79	0.20	**−** 1.10, 1.50
Mediators on ASD diagnosis:								
Gaze aversion (*a*_1_)	6.07	0.77, 11.37	6.07	0.77, 11.37	6.07	0.77, 11.37	6.07	0.77, 11.37
Repetitive body movements (*a*_2_)	1.92	**−** 3.06, 6.90	1.92	**−** 3.06, 6.90	1.92	**−** 3.06, 6.90	1.92	**−** 3.06, 6.90
Literal interpretation (*a*_3_)	0.13	0.01, 0.26	0.13	0.01, 0.26	0.13	0.01, 0.26	0.13	0.01, 0.26
Poor reciprocity (*a*_4_)	1.00	**−** 0.18, 2.17	1.00	**−** 0.18, 2.17	1.00	**−** 0.18, 2.17	1.00	**−** 0.18, 2.17
Clinical Impression (*a*_5_)	1.29***	**0.94, 1.63**	1.29***	**0.94, 1.63**	1.29***	**0.94, 1.63**	1.29***	**0.94, 1.63**
Outcome on mediators								
Gaze aversion (*b*_1_)	**−** 0.01	**−** 0.02, 0.01	0.01	**−** 0.02, 0.04	0.01	**−** 0.01, 0.03	0.02	**−** 0.02, 0.06
Repetitive body movements (*b*_2_)	**−** 0.01	**−** 0.03, 0.01	**−** 0.01	**−** 0.04, 0.02	0.01	**−** 0.02, 0.03	**−** 0.03	**−** 0.07, 0.01
Literal interpretation (*b*_3_)	**−** 0.39	**−** 0.81, 0.03	**−** 0.19	**−** 1.65, 1.27	0.70	**−** 0.24, 1.63	0.72	**−** 0.65, 2.08
Poor reciprocity (*b*_4_)	**−** 0.15***	**−** **0.21, −** **0.08**	**−** 0.09	**−** 0.21, 0.03	**−** 0.01	**−** 0.11, 0.09	**−** 0.15	**−** 0.32, 0.02
Clinical impression (*b*_5_)	0.16	**−** 0.09, 0.41	**−** 0.87**	**−** **1.36, −** **0.38**	**−** 0.50**	**−** **0.82, −** **0.19**	**−** 0.53	**−** 1.21, 0.15
Indirect effects								
Total indirect effect (*ab*)	**−** 0.05	**−** 0.49, 0.39	**−** 1.20**	**−** **1.94, −** **0.45**	**−** 0.50	**−** 0.96, **−** 0.04	**−** 0.68	**−** 1.51, 0.16
Gaze aversion (*a*_1_*b*_1_)	**−** 0.03	**−** 0.15, 0.08	0.05	**−** 0.14, 0.25	0.05	**−** 0.07, 0.16	0.11	**−** 0.14, 0.37
Repetitive body movements (*a*_2_*b*_2_)	**−** 0.02	**−** 0.09, 0.05	**−** 0.02	**−** 0.09, 0.05	0.02	**−** 0.05, 0.08	**−** 0.06	**−** 0.20, 0.09
Literal interpretation (*a*_3_*b*_3_)	**−** 0.05	**−** 0.11, 0.01	**−** 0.03	**−** 0.22, 0.16	0.09	**−** 0.09, 0.27	0.10	**−** 0.12, 0.31
Poor reciprocity (*a*_4_*b*_4_)	**−** 0.15	**−** 0.34, 0.05	**−** 0.09	**−** 0.23, 0.05	**−** 0.01	**−** 0.10, 0.09	**−** 0.15	**−** 0.30, 0.01
Clinical impression (*a*_5_*b*_5_)	0.20	**−** 0.11, 0.52	**−** 1.12*	**−** **1.87, −** **0.36**	**−** 0.65**	**−** **1.06, −** **0.24**	**−** 0.68	**−** 1.55, 0.18
Indices of model fit	RMSEA = .04, CFI = .78, TLI = .53		RMSEA = .04, CFI = .82, TLI = .63		RMSEA = .04, CFI = .77, TLI = .51		RMSEA = .04, CFI = .75, TLI = .47	

*RMSEA *root mean square error of approximation, *CFI *comparison fit index; *TLI *Tucker**-**Lewis index. Estimates that are significant at the .05 level are indicated in bold

*p ≤ .05, **p ≤ .01, ***p ≤ .001

To examine the interaction effect of ASD diagnosis and knowledge of ASD diagnosis on judgments of deception and credibility, we aggregated ratings of perceived deception, competence, caring, and character according to the target individual in the stimulus video (*N* = 59). We then conducted four two-way mixed ANOVAs, with ASD diagnosis and knowledge of ASD diagnosis as the independent variables, and ratings of perceived deception, competence, caring, and character as the dependent variables, respectively (as the overall multivariate effect was not of interest, three two-way mixed ANOVAs with Bonferroni correction were used for the analysis of perceived competence, caring, and character instead of a two-way mixed MANOVA). The results revealed a significant interaction effect of ASD diagnosis and knowledge of ASD diagnosis on ratings of perceived deception [*F*(1, 57) = 9.12, *p* = .004, η_p_^2^ = .14, 90% CI .03, .27] and character [*F*(1, 57) = 6.15, *p* = .05, η_p_^2^ = .10, 90% CI .01, .23]. However, there was no significant interaction effect of ASD diagnosis and knowledge of ASD diagnosis on ratings of perceived competence [*F*(1, 57) = 5.19, *p* = .08, η_p_^2^ = .08, 90% CI .01, .21] or caring [*F*(1, 57) = 0.34, *p* = 1, η_p_^2^ = .01].

## Discussion

We examined whether autistic individuals would be perceived as more deceptive and less credible than their neurotypical peers due to their demonstration of unexpected or atypical behaviors that are commonly judged as indicative of deception: specifically, gaze aversion, repetitive body movements, literal interpretation of figurative language, poor reciprocity, and flat affect.

Autistic individuals were indeed judged as more deceptive and lower on perceived competence and character compared to neurotypical individuals. To the best of our knowledge, this study is among the first to provide empirical evidence for the existence of such a relationship. Our findings contrast sharply with those of Maras, Crane, et al. (2019), who found that, when presented with video testimonies of witnesses describing a past event, mock jurors perceived autistic witnesses to be equally as credible as neurotypical witnesses. It is possible that this discrepancy may have been due to the different contexts used in the stimulus videos. First, Maras et al.’s targets were not involved in any two-way interactions. Second, unlike the videos in the present study in which the target individuals were interviewed about their involvement in stealing money, the targets in the study by Maras, Crane, et al. (2019) were asked to provide a free memory report of a simulated first aid event. Thus, the observers were unlikely to suspect the target individuals of being dishonest as there was no apparent incentive or rationale for doing so.

### The Relationship between ASD Diagnosis and Autistic Behaviors

The expected significant behavioral differences between autistic adults and neurotypical peers in gaze aversion, repetitive body movements, literal interpretation of figurative language, poor reciprocity, and flat affect were not detected. Several possible explanations exist for this unexpected finding. First, it is important to note that, while no statistically significant differences were found between groups, small effect sizes (in two instances, approaching moderate) in the hypothesized direction were detected for certain behaviors (e.g., gaze aversion, poor reciprocity, and literal interpretation of figurative language). Given the likely large variability within both the autistic and neurotypical groups, it is possible our relatively small sample of targets did not capture the “true” proportion of autistic individuals who prominently display such behavioral characteristics. Thus, these findings remain inconclusive and highlight the need for such comparisons to be repeated with a larger sample of target stimuli, recruitment of which is likely to prove difficult.

Another possible explanation is that adults on the autism spectrum may not exhibit these behaviors to either the extent or as universally as is typically proposed. Given that a large amount of research in this area has been conducted among children, it is possible that these behavioral differences may not be reflected to the same degree in the adult population (Fecteau et al., 2003; Georgiades et al., 2017). Furthermore, there is also increasing evidence in the literature that social camouflaging is prevalent among people on the autism spectrum (Cage & Troxell-Whitman, 2019; Livingston et al., 2019) and, thus, these behaviors may not be displayed consistently across all situations. Although adults on the autism spectrum are presumed to display higher levels of gaze aversion, repetitive body movements, literal interpretation of figurative language, poor reciprocity, and flat affect than the general population, there is little in the way of rigorous behavioral data to support these assumptions. Rather, these expectations are largely based on the broad diagnostic criteria for autism outlined by the DSM-5 and clinical observations of atypical behavior, which can lend themselves to overgeneralizations about how people on the autism spectrum behave.

Although ASD diagnosis did not significantly predict the presence of any individual behavior, it was a significant predictor of overall clinical impression. Though it is unclear exactly what factors led to this impression, this provides support that there was, in fact, some form of noticeable difference in the presentation of the autistic individuals that distinguished them from neurotypical comparisons.

### The Relationship between ASD Diagnosis, Autistic Behaviors, and Perceived Deception and Credibility

Although it appears that autistic individuals are more vulnerable to being judged negatively than neurotypical individuals, it remains uncertain why this is the case. However, drawing on attribution theories (Kelley, 1971; Kelley & Michela, 1980) and the expectancy-violation model (Bond et al., 1992; Burgoon, 1983), we surmise that there is something in the presentation of autistic individuals that contradicts observers’ expectations of how a truthful and credible person should behave.

It may be that this relationship is explained by other autistic behaviors that were not explored or captured in the present study. Alternatively, perhaps subtle nuances or particular combinations of behaviors cause autistic individuals to be judged negatively. This is consistent with the proposition by Sasson et al. (2017) that “negative first impressions of ASD are not founded on any one feature of expression, but rather represent an effect of subtle physical, dynamic, and auditory cues of presentation” (p. 8). Either way, the fact that participants were able to distinguish autistic individuals from neurotypical individuals in their ratings of deception and credibility suggests that there were noticeable differences between the two groups. However, further research is necessary to identify exactly what these differences are.

### Participant-Reported Cues Indicative of Deception and Low Credibility

Participants’ qualitative reasons for their impressions revealed that gaze aversion was the most frequently cited behavioral cue used in inferring both the truthfulness (28.33%) and credibility (15.93%) of the target individuals. However, despite this, gaze aversion was not a significant predictor of perceived deception or credibility after controlling for other autistic behaviors. The large variability in participant-reported cues (particularly within responses classified under “other”) suggests that there are many different behaviors that people perceive to be indicative of deception and low credibility. It was also noted that many participants did not report the use of specific behavioral cues, but rather, referred to broad presentations (e.g., “he seemed very nervous,” “he looked relaxed,” “she felt uncomfortable at times,” “she carried a calm yet confident composure,” “he seemed a little paranoid,” “body language seemed off”) or their gut-feeling about the individual (e.g., “she seems like a really nice girl,” “she seemed honest,” “he seemed like a genuine guy,” “she looked very simple and not very eloquent,” “she seemed a bit cocky and self-interested”). These responses suggest that participants may not have been fully aware of the specific factors that influenced their judgments.

### Knowledge of ASD Diagnosis

Attribution theories (Kelley & Michela, 1980) propose that, while behaviors that are normative or expected are accepted at face value, behaviors that are atypical or unexpected demand an explanation. When no obvious explanations are available, individuals begin to search for factors to which the behavior could be attributed, such as deception and low credibility. Thus, if an explanation for the behavior was available, there would be no need to attribute the behavior to deception or low credibility. Informing participants that the target individual was autistic negated the effect of ASD diagnosis on perceived deception and character. However, ASD diagnosis continued to negatively affect ratings of perceived competence. The description of autism provided to participants highlighted that “characteristics of ASD include difficulties with social interaction and nonverbal communication, repetitive behaviors, and restricted interests.” Although this reflects DSM-5 diagnostic criteria (American Psychiatric Association, 2013), inherent in this statement is the fact that autistic individuals experience impairments in certain areas of functioning. It may be that defining autism in this manner primed participants to think of autistic individuals in terms of what they can’t do, thus resulting in lower ratings of perceived competence relative to neurotypical individuals.

### Practical Implications

Our findings suggest that although people on the autism spectrum are more likely to be viewed as deceptive and lacking credibility when compared with neurotypical individuals, there may be more that contributes to these judgments than some very striking behaviors. Instead, it could be that autistic people are more likely to display a unique combination of behaviors and nuances that discriminate them from neurotypical individuals and result in unfavorable impressions. Consequently, it is important for researchers and practitioners to recognize the complex network of factors that may influence deception and credibility judgments when planning or evaluating possible interventions. For example, prior to a court trial, attorneys often guide witnesses and defendants on how to deliver a persuasive testimony (Boccaccini et al., 2005). Witnesses and defendants are often made aware of common nonverbal behaviors that may negatively affect evaluations of their testimony and are taught to better control these behaviors (Boccaccini, 2002). For instance, defendants may be instructed to maintain appropriate eye contact with the jury, as gaze aversion is commonly associated with lack of remorse (Corwin et al., 2012). Similarly, research is emerging on the effectiveness of job interview training in improving interview performance among people on the autism spectrum (e.g., Kumazaki, Muramatsu, Yoshikawa, Matsumoto, et al., 2019; Kumazaki et al., 2017; Morgan et al., 2014). These training programs are designed to assist autistic individuals in developing good communication skills, such as appropriate eye contact and facial expressions (Kumazaki, Muramatsu, Yoshikawa, Corbett, et al., 2019). Unfortunately, if autistic individuals are perceived to lack credibility as a function of their overall presentation, simply instructing them to display certain overt behaviors may not be sufficient to negate this effect (Sasson et al., 2017) unless the key behaviors can be identified. It would thus be important for future research to examine perceptions of deception and credibility toward autistic individuals in a broad range of interpersonal situations, such as forensic investigations, job interviews, and healthcare assessments, in order to better understand the factors that contribute to these impressions.

Consistent with the findings of Maras, Crane, et al. (2019), the results of our study indicated that autistic individuals were rated as less deceptive and of higher character when observers were provided with information about their ASD diagnosis. This suggests that appropriate disclosure of an ASD diagnosis, in conjunction with relevant education on autistic behaviors, may be an effective way to reduce the negative bias toward people on the autism spectrum (Maras, Crane, et al., 2019; Maras, Marshall, et al., 2019; Sasson & Morrison, 2019). However, Maras, Crane, et al. (2019) caution that research in this area is still preliminary and a better understanding of the mechanisms through which credibility judgments are formed would be necessary to determine how these principles can be applied effectively by stakeholders.

## Limitations

Our hypothesizing was stimulated by attribution theories (Kelley & Michela, 1980), which propose that perceptions of deception and low credibility arise due to the natural tendency of observers to attribute a cause to unexpected or atypical behaviors (Feldman & Chesley, 1984). The findings of the study are consistent with this hypothesis, as there was a significant interaction effect between ASD diagnosis and observers’ knowledge of the target’s diagnosis on perceptions of deception and credibility. However, as we did not directly measure attributions, no conclusions can yet be drawn regarding the underlying cognitive processes that led to participants’ judgments of deception and credibility.

Furthermore, the effect of the target behaviors on judgments of deception and credibility was only examined in one context (i.e., being questioned about involvement in taking money from a psychology lab). We speculated that if the individual was in a situation in which it was expected that the target behavior would be displayed, the presence of the target behavior would have no effect, or perhaps even the opposite effect, on judgments of deception and credibility. However, as only one context was used for the stimulus videos, this conjecture was not empirically tested.

Another limitation of the study was that other possible confounding variables were not investigated or controlled. There is some evidence that variables such as age and gender can also influence perceptions of credibility; however, these effects, in turn, appear to interact with a variety of additional factors. For example, Neal et al. (2012) found that gender differences in the perceived credibility of expert witnesses only emerged when the witnesses lacked warmth and competence, while McKimmie et al. (2004) found that it was the congruence between an expert witness’s gender and the gender orientation of their domain of expertise that predicted perceptions of credibility. Likewise, although studies have shown that female mock jurors are more likely to perceive victims of sexual abuse as more credible than male jurors (Davies & Rogers, 2009; McCauley & Parker, 2001), male jurors have been found to perceive defendants as more credible than female jurors (McCoy & Gray, 2007; Pozzulo et al., 2010). It is thus possible that the relationship between ASD diagnosis, autistic behaviors, and perceived deception and credibility may differ according to various demographic and situational factors; however, the present study’s design and scope did not permit the examination of such interactions.

## Future Research

A crucial question that still remains unanswered is: Why were autistic individuals judged as more deceptive and less credible than their neurotypical peers? We propose four possible avenues for future research that could be considered: examining groups of cues, examining nonlinear relationships, examining the “truthful prototype,” and examining expectancy violation.

### Examining Groups of Cues

Rather than examining individual behaviors in isolation, it may be useful to examine whether certain types of cues are more predictive of perceived deception and credibility than others. For example, an interaction could be broken down into its verbal content, paralinguistic cues, and nonverbal cues. By presenting various combinations of these cues to observers (e.g., using transcript only, audio only, video only, audio and video, etc.), the degree to which each of these groups of cues interact with ASD diagnosis to affect judgments of deception and credibility can be examined. Should certain cues prove to be more predictive of perceived deception and credibility than others, this may assist researchers to develop a more targeted approach in trying to identify possible behavioral mediators of the relationship between ASD diagnosis and perceived deception and credibility. Considering groups of cues as a whole would also enable researchers to better investigate the possibility that it is particular combinations of behaviors that affect perceived deception and credibility, rather than individual behaviors on their own.

### Examining Nonlinear Relationships

The assumption of the present study was that the relationship between autistic behavior and perceived deception and credibility is a linear one. However, this may not hold true for all behaviors. For example, while high levels of gaze aversion may be unexpected or atypical, so too may extremely low levels of gaze aversion. It is thus possible that there is a particular range of the behavior that is perceived to be within normal limits and that any demonstration that is above or below this threshold is considered deviant. If so, further research using nonlinear analyses may be better suited to capture these relationships.

### Examining the “Truthful Prototype”

According to the norm-violation model of veracity judgments, individuals infer deception from nonverbal behaviors that violate social standards of appropriate behavior (Levine et al., 2000). This suggests that perceived deception may arise from a violation of the “truthful prototype” as opposed to an activation of the “deceptive prototype.” While the behaviors that constitute the deceptive prototype have been fairly well documented (e.g., The Global Deception Research Team, 2006), much less is known about the behaviors associated with perceived truthfulness. Though it would be easy to assume that the truthful prototype is simply the opposite of the deceptive prototype, this may not be the case. Perhaps the way in which the truthful and deceptive prototypes are construed are qualitatively different. Alternatively, it could be that the most salient features of the truthful prototype may differ from those of the deceptive prototype (e.g., the first thing that comes to mind when asked “How can you tell when people are lying?” may not be the direct opposite of the first thing that comes to mind when asked “How can you tell when people are telling the truth?”).

The truthiness effect suggests that nonprobative information can influence the perceived truthfulness of a claim by increasing the fluency with which the information is processed: the more easily information is processed, the more likely people are to believe it to be true (Newman et al., 2012). This effect has been found with a variety of types of nonprobative information; for example, claims are more likely to be judged as trustworthy if they are presented alongside a decorative image (Newman et al., 2012), attributed to a person with a name that is easy to pronounce (Newman et al., 2014), or played with high audio quality (Newman & Schwarz, 2018). Therefore, it could be that neurotypical individuals display a constellation of behaviors that render their communication more easily processed than that of autistic individuals. Examining deviations from the truthful prototype may thus shed light on possible behavioral cues that have previously been overlooked.

### Examining Expectancy Violation

The expectancy-violation model proposes that it is not specific behaviors per se that lead to perceptions of deception and low credibility, but rather, the fact that these behaviors are unexpected (Bond et al., 1992; Burgoon et al., 1989). Consequently, the extent to which a particular behavior is considered to be indicative of deception or low credibility may differ from observer to observer, depending on their own unique expectations of how the target individual should behave. To test this hypothesis, researchers have measured observers’ expectations and examined whether the effect of nonverbal behavior on perceived deception or credibility was influenced by the degree to which their expectations were violated (e.g., Ask & Landström, 2010; Bosma et al., 2018; Hackett et al., 2008). The findings suggest that nonverbal behaviors have stronger effects on judgments of deception and credibility when they are more incongruent with the observer’s expectations (Ask & Landström, 2010; Bosma et al., 2018; Hackett et al., 2008). It is thus possible that the reason why only small effects of autistic behaviors were detected in the present study was that the specific cues that were considered to be indicative of deception and low credibility varied for each observer and, therefore, these effects were not detected when overall mean values for specific behaviors were examined.

## Conclusions

To our knowledge, this is the first study to examine whether the demonstration of stereotypical autistic behaviors causes autistic individuals to be perceived as more deceptive and less credible than their neurotypical peers. As predicted, autistic individuals were perceived to be more deceptive and less credible relative to neurotypical individuals when telling the truth. However, limited support was found for the hypothesized pathways by which this relationship was thought to occur. No significant differences in levels of gaze aversion, repetitive body movements, literal interpretation of figurative language, poor reciprocity, or flat affect were detected between the autistic and neurotypical groups, although some weak effects were apparent. Furthermore, independent of ASD diagnosis, none of the target autistic behaviors significantly predicted judgments of deception or credibility. Instead, the results appear to suggest that an individual’s overall presentation as having a condition or disability had a stronger influence on the relationship between ASD diagnosis and perceived deception and credibility than individual behavioral cues. It is possible that this could be due to the presence of other autistic behaviors that were not accounted for in the present study, or to the complex interaction of multiple different behaviors and idiosyncrasies. Nonetheless, the findings of this study seem to indicate that there are noticeable differences in the presentation of autistic individuals that distinguish them from neurotypical individuals and result in less favorable impressions. Further research is thus necessary to better understand the exact mechanism by which this relationship occurs.

## Supplementary Information

Below is the link to the electronic supplementary material.

Supplementary file1 (DOCX 60 kb)
