# Supplementary material for: Autistic Adults May Be Erroneously Perceived as Deceptive and Lacking Credibility
Source: J Autism Dev Disord. 2021 Mar 17;52(2):490–507. doi: 10.1007/s10803-021-04963-4 (PMC8813809; doi:10.1007/s10803-021-04963-4)
Supplement: Supplementary file 1 — Supplementary file1 (DOCX 60 kb) [file 10803_2021_4963_MOESM1_ESM.docx]

**Demographics of the Target Individuals**

**Table 1**

*Demographics of the Neurotypical and Autistic Target Individuals*

| Neurotypical Individuals | | | |  | Autistic Individuals | | | |
| --- | --- | --- | --- | --- | --- | --- | --- | --- |
| No. | Gender | Age | Ethnicity |  | No. | Gender | Age | Ethnicity |
| 1 | Female | 20 | Asian |  | 30 | Female | 24 | Caucasian |
| 2 | Female | 20 | Asian |  | 31 | Male | 55 | Caucasian |
| 3 | Female | 20 | Asian |  | 32 | Male | 24 | Caucasian |
| 4 | Male | 20 | Asian |  | 33 | Male | 27 | Caucasian |
| 5 | Female | 24 | Asian |  | 34 | Female | 28 | Caucasian |
| 6 | Female | 18 | Caucasian |  | 35 | Male | 29 | Caucasian |
| 7 | Male | 27 | Caucasian |  | 36 | Male | 65 | Caucasian |
| 8 | Male | 23 | Caucasian |  | 37 | Female | 37 | Caucasian |
| 9 | Female | 20 | Asian |  | 38 | Male | 46 | Caucasian |
| 10 | Female | 21 | Caucasian |  | 39 | Male | 23 | Caucasian |
| 11 | Male | 31 | Caucasian |  | 40 | Male | 33 | Caucasian |
| 12 | Female | 22 | Asian |  | 41 | Male | 66 | Caucasian |
| 13 | Male | 23 | Caucasian |  | 42 | Male | 36 | Caucasian |
| 14 | Male | 21 | Asian |  | 43 | Female | 24 | Caucasian |
| 15 | Female | 20 | Caucasian |  | 44 | Male | 23 | Caucasian |
| 16 | Male | 28 | Asian |  | 45 | Male | 21 | Caucasian |
| 17 | Female | 21 | Caucasian |  | 46 | Male | 31 | Caucasian |
| 18 | Female | 28 | Caucasian |  | 47 | Male | 27 | Caucasian |
| 19 | Female | 38 | Caucasian |  | 48 | Male | 25 | Caucasian |
| 20 | Male | 38 | Caucasian |  | 49* | Male | 38 | Caucasian |
| 21 | Male | 32 | Caucasian |  | 50 | Male | 26 | Caucasian |
| 22 | Male | 25 | Caucasian |  | 51 | Male | 42 | Caucasian |
| 23 | Female | 23 | Caucasian |  | 52 | Female | 62 | Caucasian |
| 24 | Male | 22 | Asian |  | 53 | Male | 22 | Caucasian |
| 25 | Male | 24 | Caucasian |  | 54 | Male | 26 | Caucasian |
| 26 | Male | 24 | Caucasian |  | 55 | Female | 19 | Caucasian |
| 27 | Female | 21 | Caucasian |  | 56 | Male | 36 | Caucasian |
| 28 | Male | 36 | Caucasian |  | 57 | Male | 30 | Caucasian |
| 29 | Female | 25 | Caucasian |  | 58 | Female | 18 | Caucasian |
|  |  |  |  |  | 59 | Female | 48 | Caucasian |
|  |  |  |  |  | 60 | Female | 51 | Caucasian |

*The video from this individual was excluded from the study as the truthfulness of his responses could not be ascertained.

**Measure of Source Credibility**

Participants were asked to rate the perceived credibility of each target individual using a modified version of McCroskey and Teven’s (1999) measure of source credibility. This measure of source credibility comprises three 6-item dimensions: Competence, Caring, and Character. McCroskey and Teven's (1999) validation study revealed that each of the three dimensions accounted for significant variance as unique predictors of believability and likableness, and the authors argue that, rather than sum the scores of all items in the measure to form one measurement of source credibility, the use of all three individual dimensions in the operationalization of source credibility would be more appropriate.

Items on this measure were rated on a 7-point semantic differential scale, with higher scores indicating higher levels of perceived credibility. Sample items include “incompetent/competent,” “self-centered/not self-centered,” and “untrustworthy/trustworthy.” Three items within the Caring subscale were adapted to improve the relevance of the items to the present context (e.g., “cares about me/doesn’t care about me,” was modified to “cares about the interviewer/doesn’t care about the interviewer”). The instructions for completing the measure were also slightly modified to better suit the purpose of the present study: “Please indicate your impression of the person noted below by circling the appropriate number between the pairs of adjectives below. The closer the number is to an adjective, the more certain you are of your evaluation,” was modified to “Please indicate your impression of the person in the interview by selecting the appropriate point between the pairs of adjectives below. The closer the number is to an extreme, the more accurate you consider that description to be.”

To test the model fit, confirmatory factor analyses (CFA) using maximum likelihood (ML) estimation were carried in a prior study (Lim, 2020). The results indicated that the hypothesized three-factor model was a poor fit for the data, and thus, respecification of the model was pursued through post hoc adjustments. The resulting best-fit model consisted of five items from the Competence subscale, three items from the Caring subscale, and six items from the Character subscale (see Table 2). This model was therefore used as the basis for the operationalization of perceived competence, caring, and character for this study.

**Table 2**

*Operationalization of Perceived Competence, Caring, and Character*

| Variable | Operationalization |
| --- | --- |
| Perceived Competence | The sum of scores on items 1, 3, 4, 5, and 6 of the Competence subscale of McCroskey & Teven’s (1999) measure of source credibility, with higher scores indicating higher levels of perceived competence. |
| Perceived Caring | The sum of scores on items 1, 2, and 4 of the Caring subscale of McCroskey & Teven’s (1999) measure of source credibility, with higher scores indicating higher levels of perceived caring. |
| Perceived Character | The sum of scores on all items of the Character subscale of McCroskey & Teven’s (1999) measure of source credibility, with higher scores indicating higher levels of perceived character. |

**Individual Multilevel Mediation Models**

**Table 3**

*Individual Multilevel Mediation Models between ASD Diagnosis, Autistic Behaviors, and Perceived Deception and Credibility*

|  | Perceived Deception | | Perceived Competence | | Perceived Caring | | Perceived Character | |
| --- | --- | --- | --- | --- | --- | --- | --- | --- |
|  | Estimate | 95% CI | Estimate | 95% CI | Estimate | 95% CI | Estimate | 95% CI |
| Gaze Aversion |  |  |  |  |  |  |  |  |
| Total Effect (*c*) | **0.86**** | **0.39, 1.32** | **-2.48***** | **-3.52, -1.43** | -0.58 | -1.18, 0.01 | **-1.88*** | **-3.31, -0.45** |
| Direct Effects |  |  |  |  |  |  |  |  |
| Mediator on ASD Diagnosis (*a*) | 6.07 | 0.77, 11.37 | 6.07 | 0.77, 11.37 | 6.07 | 0.77, 11.37 | 6.07 | 0.77, 11.37 |
| Outcome on Mediator (*b*) | -0.01 | -0.03, 0.01 | -0.03 | -0.07, 0.02 | -0.01 | -0.03, 0.01 | -0.04 | -0.10, 0.02 |
| Outcome on ASD Diagnosis (*c’*) | **0.93**** | **0.43, 1.42** | **-2.31***** | **-3.38, -1.24** | -0.51 | -1.09, 0.07 | -1.63 | -3.06, -0.20 |
| Indirect Effect (*ab*) | -0.07 | -0.19, 0.05 | -0.17 | -0.48, 0.15 | -0.07 | -0.21, 0.07 | -0.25 | -0.64, 0.15 |
|  |  |  |  |  |  |  |  |  |
| Repetitive Body Movements |  |  |  |  |  |  |  |  |
| Total Effect (*c*) | **0.85**** | **0.39, 1.32** | **-2.48***** | **-3.52, -1.43** | -0.58 | -1.17, 0.01 | **-1.88*** | **-3.31, -0.45** |
| Direct Effects |  |  |  |  |  |  |  |  |
| Mediator on ASD Diagnosis (*a*) | 1.92 | -3.06, 6.90 | 1.92 | -3.06, 6.90 | 1.92 | -3.06, 6.90 | 1.92 | -3.06, 6.90 |
| Outcome on Mediator (*b*) | -0.02 | -0.04, 0.00 | -0.02 | -0.06, 0.01 | 0.03 | 0.00, 0.05 | 0.001 | -0.06, 0.06 |
| Outcome on ASD Diagnosis (*c’*) | **0.89***** | **0.44, 1.34** | **-2.43***** | **-3.49, -1.38** | -0.63 | -1.21, -0.04 | **-1.88*** | **-3.28, -0.48** |
| Indirect Effect (*ab*) | -0.04 | -0.13, 0.06 | -0.05 | -0.19, 0.10 | 0.05 | -0.10, 0.19 | 0.001 | -0.11, 0.11 |
|  |  |  |  |  |  |  |  |  |
| Literal Interpretation of Figurative Language |  |  |  |  |  |  |  |  |
| Total Effect (*c*) | **0.85**** | **0.39, 1.32** | **-2.47***** | **-3.52, -1.43** | -0.58 | -1.18, 0.01 | **-1.88*** | **-3.31, -0.45** |
| Direct Effects |  |  |  |  |  |  |  |  |
| Mediator on ASD Diagnosis (*a*) | 0.13 | 0.01, 0.26 | 0.13 | 0.01, 0.26 | 0.13 | 0.01, 0.26 | 0.13 | 0.01, 0.26 |
| Outcome on Mediator (*b*) | -0.33 | -1.00, 0.34 | 0.99 | -0.43, 2.41 | **0.96*** | **0.25, 1.68** | 2.08 | -0.001, 4.16 |
| Outcome on ASD Diagnosis (*c’*) | **0.90**** | **0.42, 1.37** | **-2.60***** | **-3.73, -1.48** | -0.71 | -1.33, -0.09 | **-2.15*** | **-3.69, -0.62** |
| Indirect Effect (*ab*) | -0.04 | -0.14, 0.05 | 0.13 | -0.12, 0.38 | 0.13 | -0.06, 0.31 | 0.28 | -0.19, 0.74 |
|  |  |  |  |  |  |  |  |  |
|  |  |  |  |  |  |  |  |  |
|  |  |  |  |  |  |  |  |  |
| Poor Reciprocity |  |  |  |  |  |  |  |  |
| Total Effect (*c*) | **0.86**** | **0.40, 1.33** | **-2.48***** | **-3.52, -1.43** | -0.59 | -1.18, 0.01 | **-1.89*** | **-3.31, -0.46** |
| Direct Effects |  |  |  |  |  |  |  |  |
| Mediator on ASD Diagnosis (*a*) | 1.00 | -0.18, 2.17 | 1.00 | -0.18, 2.17 | 1.00 | -0.18, 2.17 | 1.00 | -0.18, 2.17 |
| Outcome on Mediator (*b*) | **-0.07*** | **-0.13, -0.02** | -0.12 | -0.33, 0.09 | -0.08 | -0.16, -0.01 | -0.22 | -0.44, 0.01 |
| Outcome on ASD Diagnosis (*c’*) | **0.94**** | **0.45, 1.42** | **-2.36***** | **-3.43, -1.29** | -0.50 | -1.09, 0.08 | -1.67 | -3.15, -0.19 |
| Indirect Effect (*ab*) | -0.07 | -0.16, 0.02 | -0.12 | -0.39, 0.15 | -0.08 | -0.22, 0.05 | -0.22 | -0.58, 0.15 |
|  |  |  |  |  |  |  |  |  |
| Flat Affect |  |  |  |  |  |  |  |  |
| Total Effect (*c*) | **0.86**** | **0.39, 1.33** | **-2.47***** | **-3.52, -1.42** | -0.58 | -1.17, 0.02 | **-1.87*** | **-3.30, -0.44** |
| Direct Effects |  |  |  |  |  |  |  |  |
| Mediator on ASD Diagnosis (*a*) | 0.06 | -0.08, 0.21 | 0.06 | -0.08, 0.21 | 0.06 | -0.08, 0.21 | 0.06 | -0.08, 0.21 |
| Outcome on Mediator (*b*) | 0.14 | -0.79, 1.06 | -1.14 | -2.58, 0.30 | **-1.19**** | **-1.91, -0.46** | -2.20 | -4.74, 0.35 |
| Outcome on ASD Diagnosis (*c’*) | **0.85**** | **0.37, 1.33** | **-2.40***** | **-3.46, -1.33** | -0.50 | -1.07, 0.07 | **-1.73*** | **-3.16, -0.30** |
| Indirect Effect (*ab*) | 0.01 | -0.06, 0.08 | -0.07 | -0.30, 0.15 | -0.08 | -0.26, 0.11 | -0.14 | -0.56, 0.28 |
|  |  |  |  |  |  |  |  |  |
| Clinical Impression of ASD |  |  |  |  |  |  |  |  |
| Total Effect (*c*) | **0.86**** | **0.39, 1.33** | **-2.48***** | **-3.53, -1.43** | -0.59 | -1.18, 0.01 | **-1.89*** | **-3.32, -0.47** |
| Direct Effects |  |  |  |  |  |  |  |  |
| Mediator on ASD Diagnosis (*a*) | **0.89***** | **0.57, 1.21** | **0.89***** | **0.57, 1.21** | **0.89***** | **0.57, 1.21** | **0.89***** | **0.57, 1.21** |
| Outcome on Mediator (*b*) | -0.01 | -0.32, 0.30 | **-0.90*** | **-1.58, -0.21** | -0.37 | -0.75, 0.002 | **-1.41*** | **-2.41, -0.41** |
| Outcome on ASD Diagnosis (*c’*) | **0.87*** | **0.30, 1.44** | **-1.68*** | **-2.84, -0.52** | -0.25 | -0.89, 0.38 | -0.63 | -2.42, 1.16 |
| Indirect Effect (*ab*) | -0.01 | -0.29, 0.27 | -0.80 | -1.49, -0.11 | -0.33 | -0.69, 0.02 | **-1.26*** | **-2.28, -0.24** |
|  |  |  |  |  |  |  |  |  |
| Clinical Impression of Any Disorder |  |  |  |  |  |  |  |  |
| Total Effect (*c*) | **0.87**** | **0.40, 1.34** | **-2.49***** | **-3.53, -1.44** | -0.59 | -1.18, 0.003 | **-1.90*** | **-3.32, -0.48** |
| Direct Effects |  |  |  |  |  |  |  |  |
| Mediator on ASD Diagnosis (*a*) | **1.29***** | **0.94, 1.63** | **1.29***** | **0.94, 1.63** | **1.29***** | **0.94, 1.63** | **1.29***** | **0.94, 1.63** |
| Outcome on Mediator (*b*) | 0.17 | -0.12, 0.46 | **-1.36**** | **-2.07, -0.65** | **-0.58**** | **-0.88, -0.27** | **-1.92***** | **-2.68, -1.16** |
| Outcome on ASD Diagnosis (*c’*) | 0.65 | 0.08, 1.21 | -0.74 | -2.02, 0.55 | 0.15 | -0.52, 0.82 | 0.57 | -1.11, 2.24 |
| Indirect Effect (*ab*) | 0.22 | -0.16, 0.60 | **-1.75*** | **-2.89, -0.61** | **-0.74**** | **-1.18, -0.30** | **-2.47***** | **-3.62, -1.31** |

*Note.* Estimates that are significant at the .05 level are indicated in bold.

**p* ≤ .05, ***p* ≤ .01, ****p* ≤ .001

**Multilevel Mediation Models with Flat Affect as a Mediator**

**Table 4**

*Multilevel Mediation Models between ASD Diagnosis, Autistic Behaviors, and Perceived Deception and Credibility (with Flat Affect)*

|  | Perceived Deception | | Perceived Competence | | Perceived Caring | | Perceived Character | |
| --- | --- | --- | --- | --- | --- | --- | --- | --- |
|  | Estimate | 95% CI | Estimate | 95% CI | Estimate | 95% CI | Estimate | 95% CI |
| *Total Effects* |  |  |  |  |  |  |  |  |
| ASD Diagnosis (*c*) | **0.87**** | **0.40, 1.34** | **-2.49***** | **-3.54, -1.44** | -0.58 | -1.17, 0.01 | **-1.91*** | **-3.33, -0.49** |
|  |  |  |  |  |  |  |  |  |
| *Direct Effects* |  |  |  |  |  |  |  |  |
| ASD Diagnosis (*c’*) | 0.64 | 0.10, 1.18 | -0.77 | -2.13, 0.59 | -0.05 | -0.79, 0.69 | 0.33 | -1.48, 2.14 |
|  |  |  |  |  |  |  |  |  |
| *Mediators on ASD Diagnosis:* |  |  |  |  |  |  |  |  |
| Gaze Aversion (*a_1_*) | 6.07 | 0.77, 11.37 | 6.07 | 0.77, 11.37 | 6.07 | 0.77, 11.37 | 6.07 | 0.77, 11.37 |
| Repetitive Body Movements (*a_2_*) | 1.92 | -3.06, 6.90 | 1.92 | -3.06, 6.90 | 1.92 | -3.06, 6.90 | 1.92 | -3.06, 6.90 |
| Literal Interpretation (*a_3_*) | 0.13 | 0.01, 0.26 | 0.13 | 0.01, 0.26 | 0.13 | 0.01, 0.26 | 0.13 | 0.01, 0.26 |
| Poor Reciprocity (*a_4_*) | 1.00 | -0.18, 2.17 | 1.00 | -0.18, 2.17 | 1.00 | -0.18, 2.17 | 1.00 | -0.18, 2.17 |
| Flat Affect (*a_5_*) | 0.06 | -0.08, 0.21 | 0.06 | -0.08, 0.21 | 0.06 | -0.08, 0.21 | 0.06 | -0.08, 0.21 |
| Clinical Impression (*a_6_*) | **1.29***** | **0.94, 1.63** | **1.29***** | **0.94, 1.63** | **1.29***** | **0.94, 1.63** | **1.29***** | **0.94, 1.63** |
|  |  |  |  |  |  |  |  |  |
| *Outcome on Mediators:* |  |  |  |  |  |  |  |  |
| Gaze Aversion (*b_1_*) | -0.01 | -0.03, 0.01 | -0.002 | -0.04, 0.04 | -0.01 | -0.03, 0.01 | -0.01 | -0.06, 0.04 |
| Repetitive Body Movements (*b_2_*) | **-0.03*** | **-0.05, -0.01** | -0.03 | -0.07, 0.01 | 0.02 | -0.01, 0.04 | -0.01 | -0.07, 0.04 |
| Literal Interpretation (*b_3_*) | -0.12 | -0.64, 0.40 | 0.99 | -0.68, 2.66 | 0.63 | -0.13, 1.40 | 1.80 | -0.22, 3.82 |
| Poor Reciprocity (*b_4_*) | **-0.11**** | **-0.17, -0.04** | -0.07 | -0.25, 0.10 | -0.03 | -0.09, 0.04 | -0.12 | -0.26, 0.03 |
| Flat Affect (*b_5_*) | -0.46 | -1.56, 0.65 | 0.34 | -1.32, 2.00 | -0.43 | -1.29, 0.43 | 0.10 | -2.60, 2.79 |
| Clinical Impression (*b_6_*) | 0.37 | 0.05, 0.68 | **-1.34*** | **-2.31, -0.38** | -0.42 | -0.82, -0.02 | **-1.77***** | **-2.67, -0.86** |
|  |  |  |  |  |  |  |  |  |
| *Indirect Effects* |  |  |  |  |  |  |  |  |
| Total Indirect Effect (*ab*) | 0.23 | -0.19, 0.65 | **-1.72*** | **-3.01, -0.43** | -0.53 | -1.07, 0.01 | **-2.24**** | **-3.49, -0.98** |
| Gaze Aversion (*a_1_b_1_*) | -0.04 | -0.18, 0.09 | -0.02 | -0.27, 0.24 | -0.06 | -0.20, 0.08 | -0.07 | -0.38, 0.24 |
| Repetitive Body Movements (*a_2_b_2_*) | -0.05 | -0.19, 0.09 | -0.06 | -0.23, 0.11 | 0.04 | -0.08, 0.15 | -0.02 | -0.14, 0.10 |
| Literal Interpretation (*a_3_b_3_*) | -0.02 | -0.08, 0.05 | 0.13 | -0.14, 0.40 | 0.08 | -0.06, 0.23 | 0.24 | -0.18, 0.66 |
| Poor Reciprocity (*a_4_b_4_*) | -0.11 | -0.23, 0.02 | -0.07 | -0.26, 0.12 | -0.03 | -0.09, 0.04 | -0.12 | -0.30, 0.07 |
| Flat Affect (*a_5_b_5_*) | -0.03 | -0.09, 0.03 | 0.02 | -0.08, 0.12 | -0.03 | -0.12, 0.06 | 0.01 | -0.16, 0.17 |
| Clinical Impression (*a_6_b_6_*) | 0.47 | 0.05, 0.89 | **-1.73*** | **-3.14, -0.31** | -0.54 | -1.07, -0.01 | **-2.27**** | **-3.47, -1.08** |
|  |  |  |  |  |  |  |  |  |
| Indices of Model Fit | RMSEA = .06, CFI = .56, TLI = .18 | | RMSEA = .06, CFI = .62, TLI = .30 | | RMSEA = .06, CFI = .59, TLI = .23 | | RMSEA = .06, CFI = .59, TLI = .24 | |
|  |  |  |  |  |  |  |  |  |

*Note.* Estimates that are significant at the .05 level are indicated in bold.

**p* ≤ .05, ***p* ≤ .01, ****p* ≤ .001

**References**

Lim, A. (2020). *The effect of autism spectrum disorder behaviours on perceived deception and credibility* (Doctoral dissertation, Flinders University, Adelaide, Australia). Retrieved from https://flinders-primo.hosted.exlibrisgroup.com/permalink/f/oh7459/FUL_ALMA11177013660001771
